# First-in-Human Evaluation of the Safety and Efficacy of a Novel Stent Positioning Assistance System for Precise Positioning of Coronary Stents

**DOI:** 10.1155/2022/1683309

**Published:** 2022-03-27

**Authors:** Elias Hellou, Michael Jonas, Danny Dvir

**Affiliations:** ^1^Cardiovascular Department, E.M.M.S. Hospital, Nazareth, Israel, and Technion - Israeli Institute of Technology, Haifa, Israel; ^2^HeartInstitute, Kaplan Medical Center, Rehovot, Hebrew University, Jerusalem, Israel; ^3^Interventional Cardiology, Jesselson Integrated Heart Center, ShaareZedek Medical Center, Hebrew University, Jerusalem, Israel

## Abstract

**Objectives:**

This study was planned for evaluating the safety and efficacy of SPAS (stent positioning assistance system) device in first-in-human procedures.

**Background:**

SPAS is a novel device that can be used for improved positioning of coronary stents.

**Methods:**

Consecutive patients underwent percutaneous coronary intervention (PCI) with the SPAS device. Device-related adverse and serious adverse events were evaluated in addition to a dedicated questionnaire completed by operators immediately after using SPAS.

**Results:**

The SPAS device was deployed in 55 PCI procedures, comprising of heavily calcified lesions (33.3%), totally occluded (7.4%), and severely tortuous vessels (7.4%). In these procedures, nonbifurcation and nonostial (53.7%), bifurcation (22.2%), and edge-to-edge (24.1%) stenting techniques were employed. Analysis of the pooled scores for the five satisfaction-related questions gave an average score of 5.6 ± 1.5, with 40 (75.5%) operators providing an average satisfaction grade of >5; the average operator-rated SPAS device accuracy performance scores exceeded 6 out of 7 (on visual analog score). The time spent for positioning the stent with the SPAS device averaged 41 ± 68.0 seconds. The SPAS device was rated as easy to use (6.1 ± 1.6) and reliable (6.1 ± 1.7). No device-related adverse events were reported.

**Conclusion:**

This stent positioning device was evaluated in a consecutive cohort of standard and complex PCI procedures. The device was shown to be safe, easy, and precise to use, both in standard and complex cases.

## 1. Introduction

Despite ongoing innovations in stent delivery systems, the accuracy of stent positioning remains a significant challenge in clinical practice. Geographic miss (GM) is a descriptive terminology for a poorly deployed coronary stent or a stent missing a lesion during a percutaneous coronary intervention (PCI) procedure. GM includes longitudinal (injured or diseased segment not covered by the stent) or axial GM (balloon-artery size ratio <0.9 or >1.3) mismatches [1]. A longitudinal miss is generally a technical failure (interventionist error) that can result in edge lesions (either stent inflow or outflow lesions), while an axial miss is often a design or concept failure potentially leading to in-stent lesions [2].

Longitudinal GM has been reported to occur in up to 30–45% of PCI procedures, despite satisfactory angiographic stent positioning [1, 3]. It has been associated with an increased number of stents placed per procedure, overlapping stents, increased target vessel revascularization, myocardial infarction (MI), in-stent thrombosis, and death [2,4,5]. Additionally, it is also can lead to a higher cost of the procedure. Moreover, plaques exhibiting GM are associated with an increased percentage of necrotic core and dense calcium and are more likely to have thin-cap fibroatheroma, that is, vulnerable plaque [3].

Current heart catheterization rooms are equipped with enhanced X-ray systems that enable the interventional cardiologists to see precise images, high quality, and gated fluoroscopy and intravascular ultrasound (IVUS)/optical coherence tomography (OCT) [6]. Additional X-ray-based add-on is the SyncVision® System (Philips Volcano) image processing system with unique enhancement and stabilization power of the stent deployment system in the moving coronary artery bed, which can show the device-motion indication of the stent in relation to the desired PCI area [7]. According to previous studies [7], the median axial displacement is 2.97 mm at the right coronary artery, 2.22 mm at the left circumflex, and 1.84 mm at the left anterior descending segments. The most dynamic segments were the mid and distal right coronary artery. Both heart rate and cardiac contractility significantly affect stent movement, jeopardizing precise stent deployment.

There is a clear need for precise stent positioning, and avoiding GM.Ostial Pro® (Merit Medical Systems) enables more accurate stent placement, limited to aorto-ostial lesions [8]. Rapid ventricular pacing is believed to reduce heart motion and facilitate stent deployment to the optimal position during coronary artery stenting [9–11]. However, there is still an unmet need for stent accuracy in nonaorto-ostial lesions [5].

Robotic-assisted percutaneous coronary intervention is a growing field, mainly due to the need of implementing a more precise delivery and deployment technique for PCI devices. This intervention has the potential of reducing the occurrence of LGM [1, 12]. The high procedural costs and the need for trained personnel for operating such systems are yet significant obstacles to overcome. Robotic-assisted PCI had a significantly lower incidence of LGM compared to standard manual PCI. Reducing LGM potentially improves long-term clinical outcomes through a reduction in MACE [4]. SPAS is a universal delivery accessory that aims to enable accurate stent positioning over the entire coronary vessels. We report a first-in-human study to evaluate the safety and accuracy of SPAS to aid in that field.

## 2. Materials and Methods

### 2.1. Study Design

In this first-in-human, multicenter, cross-sectional study, adult patients planned to undergo a standard PCI procedure with stenting. The PCI procedure was performed as per standard of care, with the Stent Positioning Assistance System (SPAS) device installed on the stent delivery system (SDS) of choice. Patients were then followed up during hospitalization and once by phone seven days after hospital discharge. User feedback was obtained via a questionnaire completed by the attending interventional cardiologist. In addition, all safety-related events were documented and reviewed. Stent positioning was assessed by X-ray images/videos in all patients and by optical coherence tomography (OCT) imaging in four patients. Operator information, stent device, and procedural details were also collected. Prior to the study, participating sites obtained Institutional Ethics Committee board approval. This study was conducted in accordance with good clinical practice (GCP) guidelines.

### 2.2. Selection of Study Population

Patients underwent a PCI procedure with the SPAS device. Eligible patients were ≥18 years of age with clinically significant coronary artery stenosis. Lesions in this study were unlimited and included bifurcation lesions, ostial lesions, and edge-to-edge stenting technique. Patients with highly calcified target lesions, unstable hemodynamics, visible intravascular thrombosis, or nonvisible distal part of the target vessel were excluded. Diagnostic catheterization (referred for coronary artery bypass grafting (CABG) or nonsignificant coronary artery disease) was considered as screen failure.

### 2.3. Study Device and Interventional Procedures

SPAS is a simple device aimed to aid the interventional cardiologist in controlling the stent position in the treated artery during PCI.

**Figure 1:**
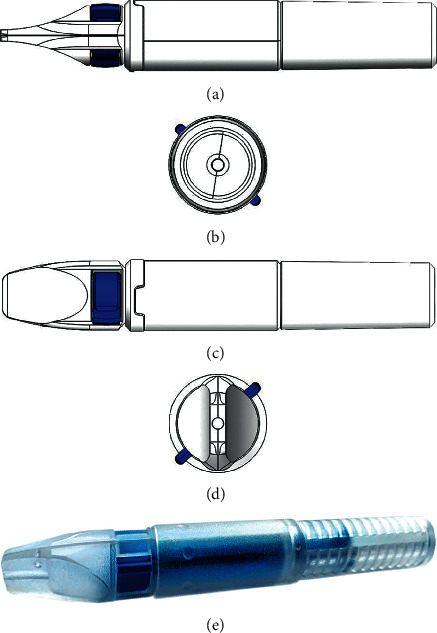
SPAS structure. (a) Top view; (b) back view; (c) side view; (d) front view; (e) 3D view of SPAS, showing the central tunnel and the locker (blue portion) and the “fixator” in the front.

**Figure 2:**
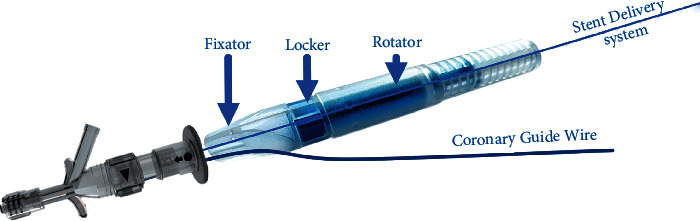
SPAS usability. SPAS support all coronary stent delivery systems. By introducing the stent from the back of the SPAS through the central main tunnel before the rapid exchange system is advanced over the coronary wire and the stent is introduced to the lesion in the coronary artery. While fixating the SPAS and the guide wire (with the thumb and index finger on the “fixator”, the “locker” is rotated until a click is felt), in order to tightly secure the SPAS on to the stent delivery system. Rather than pushing and pulling the stent inside the artery, the stent can be moved forward and backward to the accurate location by rotation of the “rotator”; clockwise drives the stent forward, and counterclockwise retracts the stent. At any point in which the operator does not feel comfortable or needs to proceed in the classic way, the “locker” is released and manual positioning can take place within seconds.

As shown in Figures 1 and 2, SPAS has a simple structure that allows installation on any coronary stent delivery system. SPAS (as described in Figure 1) is cylindrical in shape and comprises four main parts: a central channel, a site for holding the entire SPAS while moving the stent, a fixating dial, and a rotating portion. Prior to introducing the SDS on a rapid exchange system, the SPAS should be installed on the SDS by inserting the stent from the back of the SPAS into the central channel and moving it to the back of the SDS. The SDS and the SPAS are then loaded on the guide wire regularly, while the stent is pushed to an approximate desired location in the treated artery. Then, the SPAS is locked on the SDS, and the stent is moved (together with the SDS) while fixating the SPAS and the guide wire (with the thumb and index finger on the “fixator”, the “locker” is rotated until a click is felt) in order to tightly secure the SPAS on to the stent delivery system.

**Figure 3:**
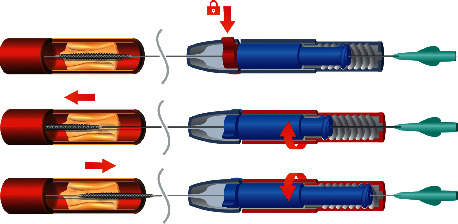
Locking and using the SPAS to move the stent to the accurate location in the artery: clockwise rotation drives the stent distally in the artery, and counter clockwise rotations retrieve the stent proximally.

By introducing the stent from the back of the SPAS through the central main tunnel before, the rapid exchange system is advanced over the coronary wire and the stent is introduced to the lesion in the coronary artery. While fixating the SPAS and the guide wire (with the thumb and index finger on the “fixator”, the “locker” is rotated until a click is felt), in order to tightly secure the SPAS on to the stent delivery system. Rather than pushing and pulling the stent inside the artery, the stent can be moved forward and backward to the desired location by rotation of the “rotator”; clockwise drives the stent forward, and counterclockwise retracts the stent as shown in Figure 3. At any point in which the operator does not feel comfortable or needs to proceed in a classic way, the “locker” is released, and manual positioning can take place within seconds.

### 2.4. Usability Measures

Anatomical features, procedural characteristics, and using SPAS in different stenting techniques were evaluated after each PCI using SPAS for stent positioning. After each PCI procedure, operators completed the “Usability Questionnaire” as appears in the supplementary files.

Operator feedback regarding usability of the SPAS device was gathered using a 7-point scale device usability questionnaire, where 7 points correspond to—“I absolutely agree”, and 1 point to—“I strongly disagree”. Success criterion was mean strong agreements with the questionnaire statements or average score >5 in each question.

### 2.5. Safety Measures

The primary safety endpoint was device-related adverse event and serious adverse event (SAE) rate during hospitalization. Adverse events (AE) were defined as any untoward medical occurrence in a study subject. Device deficiency (DD) was defined as an inadequacy of a medical device related to its identity, quality, durability, reliability, safety, or performance, such as malfunction, misuse or use error, and inadequate labeling. Adverse device effect (ADE) was defined as an AE related to the use of the investigational medical device. An SAE was defined as any untoward medical occurrence that at any dose results in death, is life-threatening, requires inpatient hospitalization or prolongation of existing hospitalization, results in persistent or significant disability/incapacity, or is a congenital anomaly/birth defect.

### 2.6. Statistical Methods

Data were analyzed by SPSS statistic software version 25. All data were summarized descriptively, with sample size, absolute, and relative frequency for categorical variables and sample size, arithmetic mean, standard deviation, and median with its 25 and 75 percentiles for continuous variables. All tests were two-tailed with a significance level of 5%.

## 3. Results

In total, 55 patients participated in the study in two centers in Israel. The vast majority of patients (81.8%) were male. The average age was 61.9 ± 10.4 years. Mean BMI was 29.5 kg/m^2^, and mean systolic blood pressure was 139 mmHg. Most common chronic diseases among subjects were hyperlipidemia (78.2%), hypertension (74.5%), diabetes mellitus (56.4%), and established previous ischemic heart disease (65.5%).

Baseline characteristics and clinical data before procedure are presented in Table 1. In total, 55 procedures were performed with the SPAS device at the two study sites, and 55questionnaires were answered regarding SPAS usability. All procedures were performed by the seven board-certified interventional cardiologists: three cardiologists with a track record of >300 PCIs per year, two cardiologists with 100–200 PCIs per year, and two cardiologists with <100 PCIs per year.

Of the 55 procedures performed, 47 (85%) were performed via radial approach, four (7.4%) lesions were totally occluded, four (7.4%) involved severe tortuosity lesions, and 18 (33.3%) were moderately to highly calcified. Balloon predilatation was performed in 19 (35.2%) procedures. The different anatomical characteristics and stent positioning/implantation approach in each procedure were indicated by the interventional cardiologists in the questionnaire.

The stenting techniques used are described in Table 2. The time spent for positioning the stent with the SPAS device averaged 41 ± 68 seconds.

### 3.1. Analysis of Usability

Supplementary Figure 1 provides an overview of the questionnaire answers. Physicians expressed high satisfaction with the device performance in most procedures (average score >6, except for edge-to-edge performance = 5.3). The physicians stated that “the SPAS device is easy to use and reliable” (average score >6) and agreed that it reduces time and simplifies stenting (average score >5). They also rather agreed to use the SPAS device for stenting in their future work (average score >5).

Operators rated SPAS accuracy performance as 6.3 ± 1.2 in procedures employing nonbifurcational and nonostial stenting techniques (29/55 procedures). The mean SPAS performance score in procedures involving a moderately or highly calcified lesion (18/55) was 6.4 ± 1.5. Device performance in bifurcational (13/55) and edge-to-edge (13/55) procedures scored 6.5 ± 0.9 and 5.3 ± 2.0, respectively. High-performance ratings of 6.8 ± 0.5 were recorded for the aorto-ostial coronary artery stenting cases. The performance in the single bifurcation multiple-stent procedure was scored 7. In conclusion, performance scores for all techniques used met the success criteria; that is, mean scores exceeded 5.

In over half of the procedures (29/55; 53.7%), operators preferred the SPAS system preinstalled on the stent delivery system (SDS), while for the remaining procedures (*n* = 22; 40.7%), operators preferred it to be at the discretion of the interventional cardiologist.

Operators generally agreed with the statement that the SPAS device reduced stent installation time (5.1 ± 1.8) and stenting complexity (5.3 ± 1.6). Ease of use and reliability scored mean 6.1 points, and operators were generally willing to use SPAS in the future (5.2 ± 1.9). Further analysis of the pooled scores for the five satisfaction-related questions gave a mean score of 5.6 ± 1.5, with 40 (75.5%) operators providing an average satisfaction grade of >5, meeting the success criteria. The time spent for positioning the stent with the SPAS device averaged 41 ± 68.0 seconds (data summarized in Table 2).

The SPAS device proved to be easy to use in PCIs for a wide range of standard techniques and for a broad range of lesion types and complexities. A 7-point scale operator-rated performance questionnaire (Supplementary Table 1) describes the operator-rated usability of and satisfaction with the SPAS device. The scores for all techniques, aside from edge-to-edge stenting, were >5, meeting the usability study endpoint. Most operators were satisfied with the SPAS device and reported “it is easy to use” and “reduced stent installation time and stenting complexity”. Success criteria (average score>5) were fulfilled.

### 3.2. Analysis of Safety

No serious adverse events related to SPAS were reported in usage in 55 patients. Two unrelated serious adverse events were reported during the study period. A 72-year-old male, heavy smoker, obese, hypertensive, with multivessel coronary artery disease, suffered from sepsis, which was later associated with a positive methicillin-susceptible *Staphylococcus aureus* blood culture, requiring prolonged hospitalization and antibiotic treatment. This event was unrelated to the SPAS device or to the study procedure. Another serious adverse event was in a 67-year-old male chronically treated with hemodialysis, admitted due to non-ST elevation myocardial infarction (NSTEMI), and treated for stenosis of the left anterior descending artery. After PCI, the patient suffered from acute pulmonary edema, requiring prolonged hospitalization and urgent hemodialysis. The patient was treated with intravenous furosemide, which led to significant improvement. The event resolved after seven days and was considered unrelated to the SPAS device and study procedure.

## 4. Discussion

This is the first-in-human evaluation of the safety and efficacy of a novel SPAS for precise positioning of coronary stents. The study included 55 different PCI procedures, from the entire spectrum of coronary intervention, including standard cases, bifurcation, ostial, calcified, and edge-to-edge stenting, and the SPAS system showed good results in safety and performer satisfaction. The unmet need for precise stent positioning in the coronary artery led to several inventions, including a robotic PCI system. The SPAS is a simple and affordable device that enables and facilitates stent placement with less than a millimeter precision.

This study is the first in the field, single-armed and designed to evaluate the first use in human.

Operators using the SPAS highly rated the performance in stenting accuracy, with the highest possible rating given to the single bifurcation and multiple-stent procedures, followed by the aorto-ostial coronary artery stenting procedures. Accuracy performance for all stenting techniques used, with the exception of edge-to-edge stenting, met the predefined success criteria (score >5).

**Figure 4:**
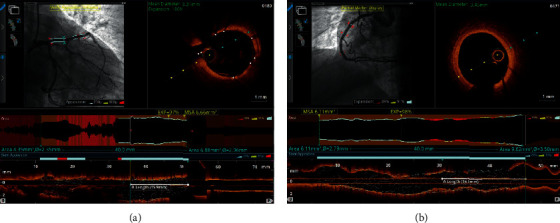
Accuracy of stents positioned with the aid of the SPAS device. Optical coherence tomography (OCT) performed following stent implantation shows (a) full correspondence between planned and actual stent position and (b) a 1 mm deviation (2.5% of stent length) from planned and actual stent position.

The performance scores were matched by OCT imaging findings performed in four patients who were randomly enrolled. Investigators decided to use this visualization method due to its high resolution and accurate length measurement. Full correspondence was found between the planned and actual implantation site in three cases and a deviation of 1 mm, that is, 2.5% of the stent length (40 mm) in one case (Figure 4). In conclusion, the GM of the stent positioning procedures performed with the SPAS device was very low and confirmed by advanced intravascular imaging.

A large group of patients (32.7%) was classified by the operators as having calcified target lesions or vessels, despite an exclusion criterion. Nevertheless, the performance scores related to these patients and procedures showed that the operators scored high in allowing the SPAS device to position the stent accurately in the lesion area.

After gaining familiarity with the SPAS device in 55 different PCI procedures, the seven interventional cardiologists who performed the stenting procedures stated that the SPAS has an “advantage in reducing stent installation time and stenting complexity”. They also indicated that the SPAS device improves the accuracy of stenting procedures. Furthermore, the SPAS was easy to use, and in most cases (66%), “it should be preinstalled on the SDS rather than leaving it to the discretion of the interventional cardiologist”.

The SPAS device proved safe when used as indicated, with no device-related adverse events reported throughout the study period.

Our study had limitations, as it is a first-in-human use of a novel device. The study is single-armed, with no control arm, reporting the experience of two centers performing PCI procedures. The interventional cardiologist received personal training for using SPAS in a bench-side PCI simulator, and the procedure evaluation was performed after the procedure. We had no control arm, and the cardiologist had to evaluate the SPAS compared with their personal experience.

Further studies emphasizing comparison of the standard of care versus the usage of SPAS system are warranted, taking into consideration stent installation time, fluoroscopy time, X-ray exposure, iodine-contrast media delivered, number of stents used, and possibly the cost of the procedure.

## 5. Conclusion

This is the first-in-human evaluation of the safety and efficacy of a novel stent positioning assistance system for precise positioning of coronary stents. The SPAS is a manual tool that brings manual PCI as close as possible to robotic-assisted PCI procedure, with a low cost and promising efficacy and safety. In our study, the SPAS device proved to be safe and easy to use with positive ratings in different PCI procedures including ostial lesions and calcified vessels. Further studies are needed for evaluating the clinical benefit of SPAS in large cohorts.

## Figures and Tables

**Table 1:** Baseline characteristics of participants (*n* = 55).

Age	61.9 ± 10.4
Male	45 (82%)
Hypertension	41(75%)
Hyperlipidemia	43 (78%)
Previous MI	11 (20%)
Diabetes mellitus	31 (56%)
Renal failure	7 (13%)
Stable ischemic heart disease	36 (65%)
Previous PCI	8 (15%)
Obesity	7 (13%)
BMI (kg/m^2^)	29.5 ± 4.5
Total occlusion	4 (8%)
Nontarget vessel with high or moderate calcium	18 (33%)

BMI: body mass index; MI: myocardial infarction; PCI: percutaneous coronary intervention.

**Table 2:** Procedural characteristics (*n* = 55).

Radial approach	47 (85%)
Balloon predilatation	19 (35%)
Heavily calcified	18 (32%)
Severe tortuosity	4 (7%)
Time spent for positioning the stent	41 ± 68.0 seconds
Target lesion	
Nonbifurcational and nonostial	29 (54%)
Edge-to-edge multiple stents	13 (24%)
Arterial bifurcation	1 (2%)
Aorto-ostial	4 (7%)
Ostial nonaorto-ostial	5 (9%)

## Data Availability

The data used to support the findings of this study are included within the supplementary information files.

## Abbreviations

AE: Adverse events
BMI: Body mass index
CABG: Coronary artery bypass grafting
GCP: Good clinical practice
GM: Geographic miss
IVUS: Intravascular ultrasound
LGM: Longitudinal geographic miss
MACE: Major adverse cardiac events
MI: Myocardial infarction
OCT: Optical coherence tomography
PCI: Percutaneous coronary intervention
SAE: Serious adverse event
SDS: Stent delivery system
SPAS: Stent positioning assistance system
SPSS: Statistical Package for the Social Sciences.

## Supplementary Materials

Supplementary Materials Supplementary Figure 1: operator-rated SPAS device accuracy performance and satisfaction in usability with the SPAS device (*n* = 55). Operators' average answer distributions of 7-point device usability SPAS experience questionnaire where 7 points “I absolutely agree” and 1 point “I strongly disagree”s. (A) Questions about ease of achieving results after performing stenting using SPAS in nonbifurcational and nonostial, ostial, bifurcational, calcified lesions, and multiple-stent edge-to-edge technique. Median score in these cases was 6.1 [5.4; 7.0]. (B) Questions about SPAS advantages in reducing time to install stent, simplifying stenting, and this device is easy to use, reliable, and willingness to use SPAS in future. Median in these questions was 5.8 [4.9; 7.0]. Supplementary Table 1: operator-rated usability of and satisfaction with the SPAS device (*n* = 55). .
