# Supplementary material for: First-in-Human Evaluation of the Safety and Efficacy of a Novel Stent Positioning Assistance System for Precise Positioning of Coronary Stents
Source: J Interv Cardiol. 2022 Mar 27;2022:1683309. doi: 10.1155/2022/1683309 (PMC8977329; doi:10.1155/2022/1683309)
Supplement: Supplementary Materials — Supplementary Figure 1: operator-rated SPAS device accuracy performance and satisfaction in usability with the SPAS device (n = 55). Operators' average answer distributions of 7-point device usability SPAS experience questionnaire where 7 points “I absolutely agree” and 1 point “I strongly disagree”s. (A) Questions about ease of achieving results after performing stenting using SPAS in nonbifurcational and nonostial, ostial, bifurcational, calcified lesions, and multiple-stent edge-to-edge technique. Median score in these cases was 6.1 [5.4; 7.0]. (B) Questions about SPAS advantages in reducing time to install stent, simplifying stenting, and this device is easy to use, reliable, and willingness to use SPAS in future. Median in these questions was 5.8 [4.9; 7.0]. Supplementary Table 1: operator-rated usability of and satisfaction with the SPAS device (n = 55). . [file 1683309.f1.docx]

**Supplementary Files**

**First-in-human Evaluation of the Safety and Efficacy of a Novel Stent Positioning Assistance System for Precise Positioning of Coronary Stents**

Elias Hellou MD^a^, Michael Jonas MD^b^, Danny Dvir MD^c^

**Supplementary Table 1**. Operator-Rated Usability of and Satisfaction with the SPAS Device (n=55)

| Question | Score Range | Mean Score |
| --- | --- | --- |
| *SPAS reduce time to install stent* | 1-7 | 5.0±1.7 |
| *SPAS simplify stenting* | 2-7 | 5.2±1.6 |
| *SPAS easy to use* | 1-7 | 6.1±1.5 |
| *SPAS is reliable* | 1-7 | 6.1±1.6 |
| *I am always ready to use SPAS in future* | 1-7 | 5.2±1.8 |

Supplementary Figure 1. Operator-Rated SPAS Device Accuracy Performance and Satisfaction in Usability with the SPAS Device (n=55)


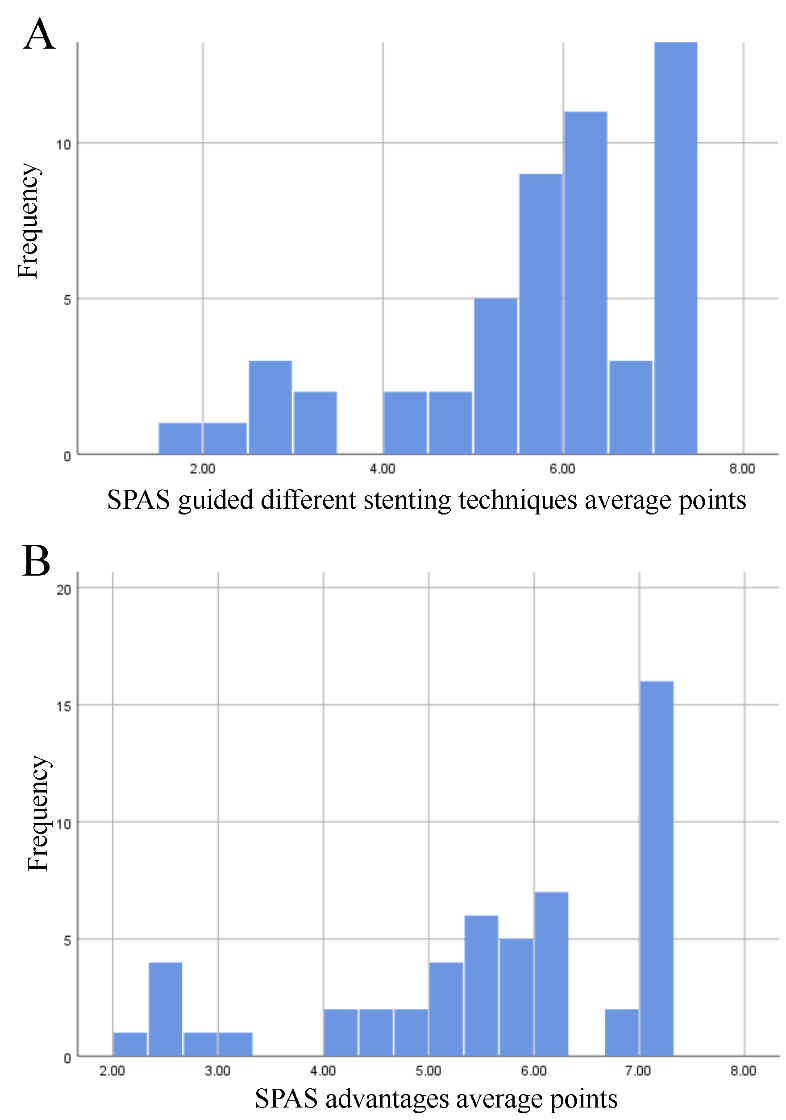


Operators’ average answer distributions of 7-point device usability SPAS experience questionnaire where 7 points – I absolutely agree, and 1 point – I strongly disagree. (A) Questions about ease of achieving results after perform stenting using SPAS in non-bifurcational and non-ostial, ostial, bifurcational, calcified lesions, multiple stent edge-to-edge technique. Median score in these cases was 6.1 [5.4;7.0]. (B) Questions about SPAS advantages in reducing time to install stent, simplifying stenting, this device is easy to use, reliable and willingness to use SPAS in future. Median in these questions was 5.8 [4.9;7.0].

**
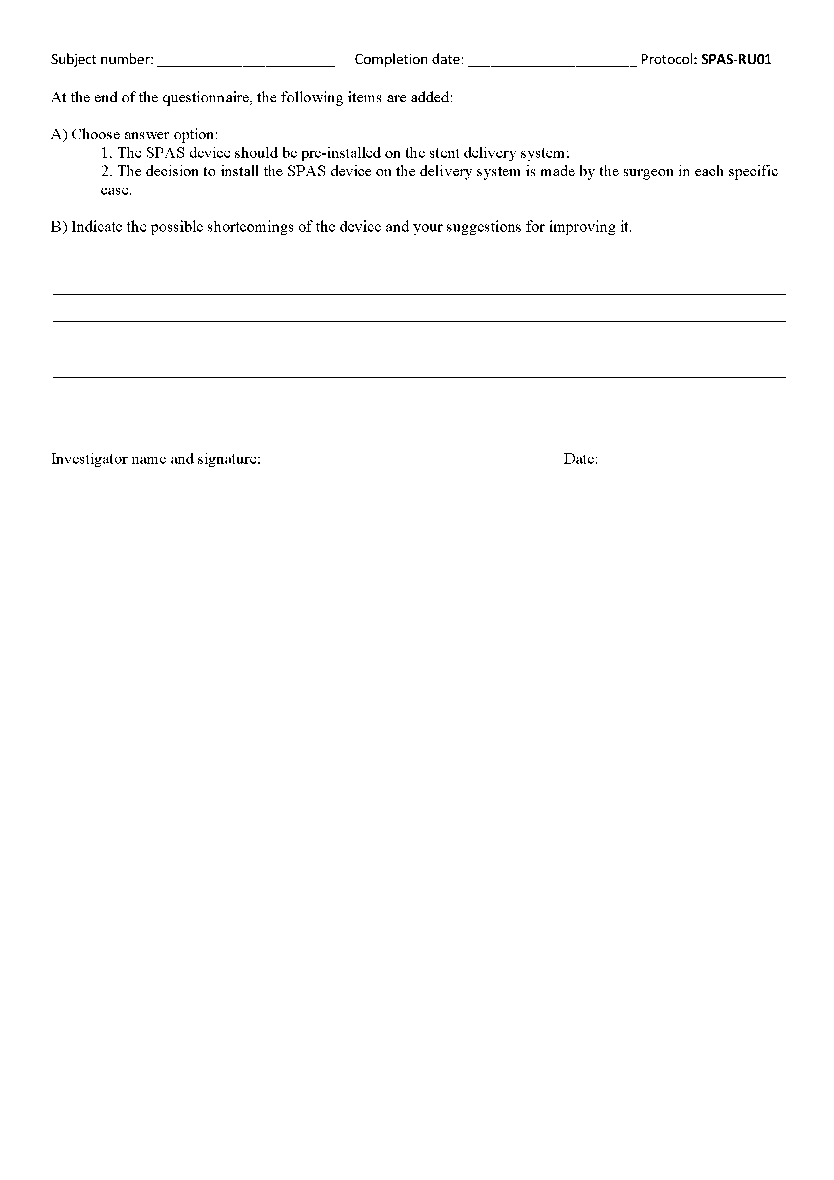

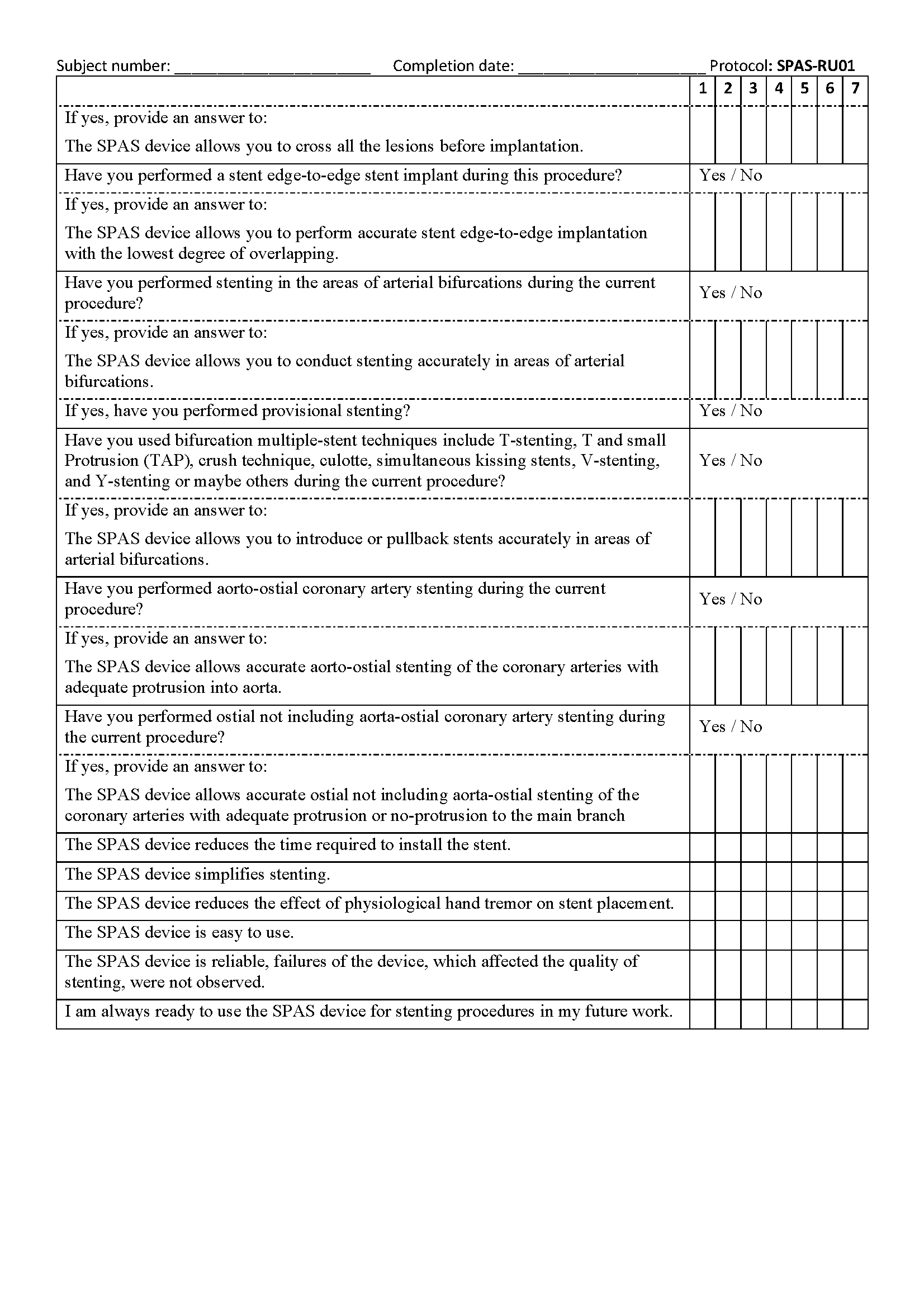

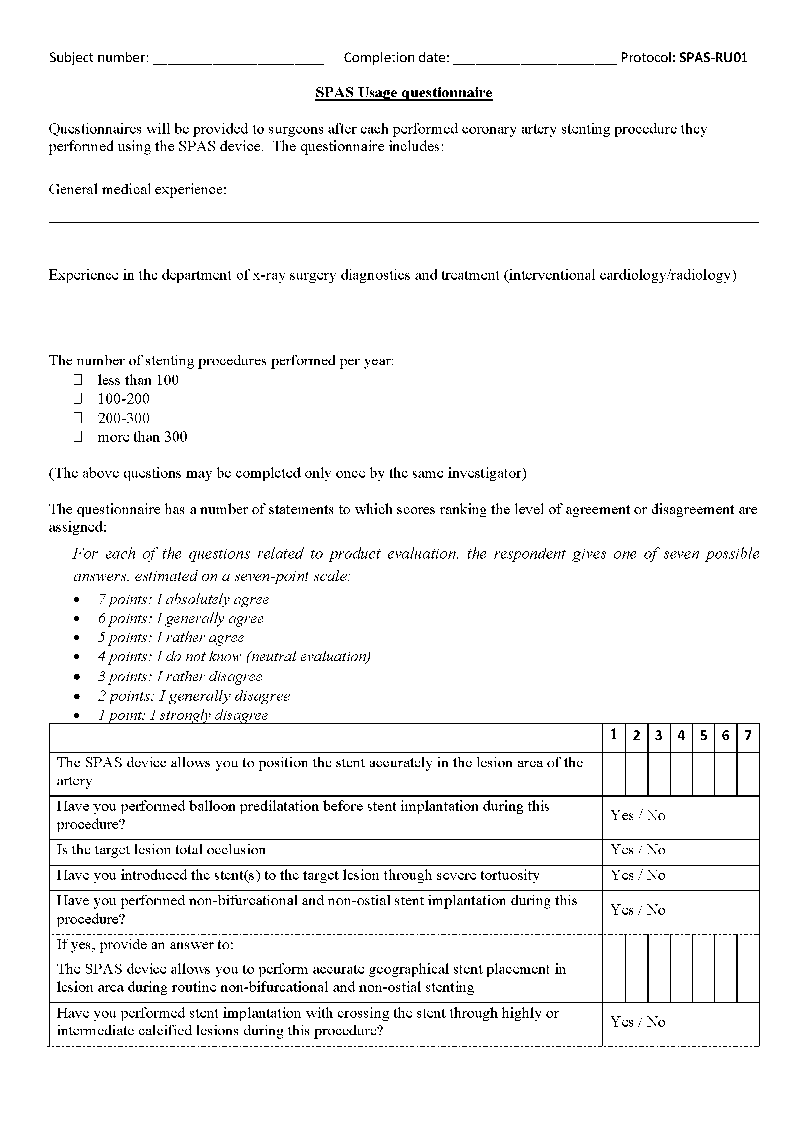
SPAS Usability Questioner**
